# Cerebrospinal fluid procalcitonin predicts Gram-negative bacterial meningitis in patients with empiric antibiotic pretreatment

**DOI:** 10.1186/s13054-019-2318-8

**Published:** 2019-01-23

**Authors:** Wen Li, Fang Yuan, Xiaolong Sun, Zhihan Zhao, Yaoyao Zhang, Wen Jiang

**Affiliations:** 1 Department of Neurology, Xijing Hospital, Fourth Military Medical University, Xi’an, 710032 People’s Republic of China; 2 Department of Rehabilitation Medicine, Xijing Hospital, Fourth Military Medical University, Xi’an, China

**Keywords:** Bacterial meningitis, Gram-negative, Procalcitonin, Diagnostic biomarker

Dear Editor,

The identification of pathogenic bacteria facilitates better targeted treatments for bacterial meningitis (BM). However, early differentiation of Gram-positive and Gram-negative BM is a persisting challenge, especially in patients with empiric antibiotic pretreatment. Serum procalcitonin (PCT) was reported to be associated with Gram-negative bacterium in bloodstream infections [1]. Our previous study demonstrated that CSF-PCT was a superior diagnostic biomarker for BM even in patients who had received empiric antibiotic pretreatment [2]. In this study, we aimed to evaluate the discriminative power of CSF-PCT for Gram-negative BM.

Consecutive patients with BM [3] admitted to neurological intensive care unit (N-ICU) at Xijing Hospital between October 2013 and April 2018 were included in this study. A total of 225 patients with suspected meningitis/encephalitis were screened. One hundred fourteen patients with tuberculous meningitis or viral meningitis/encephalitis or autoimmune encephalitis and 21 patients with unknown etiology were excluded. Ninety patients were diagnosed with BM, among them, 59 had definite pathogenic bacteria identified by CSF culture and Gram stain: 33 (56%) patients were Gram-positive, and 26 (44%) patients were Gram-negative (Additional file 1: Figure S1). Of these 59 patients, 25 (42%) cases were community-acquired and 34 (58%) cases were nosocomial BM (Additional file 1: Table S1). Bacterial etiologies were presented in Additional file 1: Table S2. All the participants had empiric antibiotic pretreatments before admission to our N-ICU. Demographics, severity, clinical characteristics, treatments, and the time from CSF-PCT test to onset of BM were balanced in Gram-positive group and Gram-negative group (Additional file 1: Table S3). The CSF-PCT level of Gram-negative patients was significantly higher than that of Gram-positive patients (0.5 [0.2–1.6] versus 0.1 [0.1–0.3] ng/ml, *p* < 0.001) (Table 1, Fig. 1). There were no significant differences of other biomarkers between Gram-positive and Gram-negative group (Additional file 1: Figure S2). CSF-PCT yielded an area under the curve (AUC) of 0.85 (95% CI, 0.73 to 0.93), which was superior to serum PCT, CSF/serum C-reactive protein (CRP) (Fig. 1).

**Table 1:** CSF and serum data

	Total (*n* = 59)	Gram-positive (*n* = 33)	Gram-negative (*n* = 26)	*P* value
CSF				
PCT, ng/mL	0.2 (0.1–0.5)	0.1 (0.1–0.3)	0.5 (0.2–1.6)	*< 0.001*
Leukocyte, cells/mm^3^	230.0 (99.0–1790.0)	214.0 (105.5–1970.0)	237.5 (56.0–1870.0)	0.54
Neutrophils, cells/mm^3^	154.1 (13.2–1432.0)	147.6 (7.0–1726.5)	165.0 (23.0–1768.9)	0.91
Lymphocyte, cells/mm^3^	50.4 (20.1–114.7)	52.1 (17.4–150.6)	44.3 (23.3–100.5)	0.71
Protein, g/L	1.6 (0.7–2.9)	1.6 (0.8–2.9)	1.6 (0.7–3.0)	0.95
Glucose, mg/dL	53.3 (37.3–72.0)	54.0 (38.9–74.7)	48.9 (34.7–69.4)	0.29
CRP, mg/L	0.3 (0.2–0.9)	0.3 (0.2–1.0)	0.3 (0.2–0.8)	0.82
First pressure, cmH_2_O	157.5 (130.0–230.0)	150.0 (127.5–205.0)	180.0 (142.5–285.0)	0.12
Last pressure, cmH_2_O	95.0 (70.0–130.0)	90.0 (70.0–128.8)	100.0 (72.5–167.5)	0.26
Serum				
PCT, ng/mL	0.6 (0.1–3.1)	0.4 (0.1–4.3)	0.8 (0.1–2.7)	0.61
Leukocyte, cells/mm^3^	11.8 (7.8–14.9)	10.5 (7.0–14.6)	13.2 (9.4–15.0)	0.16
Glucose, mg/dL	144.5 (120.6–173.8)	142.2 (117.9–175.1)	147.6 (128.5–169.7)	0.34
CRP, mg/L	9.9 (2.2–59.3)	9.5 (2.0–52.9)	14.6 (2.3–68.0)	0.71
Albumin, g/L	33.9 ± 4.4	33.1 ± 4.7	34.9 ± 4.0	0.13
CSF/serum ratio				
CSF/serum PCT ratio	0.7 (0.2–1.4)	0.7 (0.2–1.4)	0.7 (0.1–1.4)	0.75
CSF/serum glucose ratio	0.4 (0.3–0.5)	0.4 (0.3–0.5)	0.3 (0.2–0.4)	0.08

The CSF and serum samples were collected on the same day. Continuous variables were expressed as mean ± standard deviation (normally distributed), as medians and interquartile ranges (IQR, not normally distributed), which were compared using the Student *t* test or Mann-Whitney *U* test. The CSF and serum samples were collected on the same day

*CSF* cerebrospinal fluid, *PCT* procalcitonin, *CRP* C-reactive protein

**Fig. 1:**
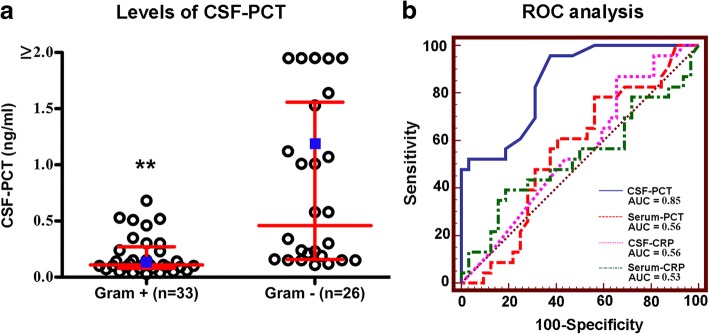
Diagnostic value of CSF-PCT for Gram-negative BM. **a** Levels of CSF-PCT in Gram-negative and Gram-positive BM. The red lines and blue squares correspond to the group interquartile ranges and averages, respectively. **P < 0.001. **b** ROC curves of CSF/serum PCT, CSF/serum CRP for predicting Gram-negative BM. CSF-PCT: AUC = 0.85 (95% CI, 0.73 to 0.93); serum PCT: AUC = 0.56 (95% CI, 0.42 to 0.69); CSF-CRP: AUC = 0.56 (95% CI, 0.42 to 0.70); serum CRP: AUC = 0.53 (95% CI, 0.39 to 0.67). Comparisons of AUC: CSF-PCT vs serum PCT, *P* < 0.0001; CSF-PCT vs CSF-CRP, *P* = 0.0034; CSF-PCT vs serum CRP, *P* = 0.0005

The early identification for the pathogenic bacteria of BM is of great importance to the rational use of antibiotics. However, meningitis with a negative Gram stain poses a diagnostic and therapeutic dilemma, especially in developing countries like China [4]. CSF and serum CRP were previously indicated to be associated with Gram-negative BM [5], but in patients with empiric antibiotic pretreatment, they were not informative any more. Our study indicated that CSF-PCT was a more robust and superior diagnostic biomarker for Gram-negative BM, even in patients who had received empiric antibiotic pretreatment.

## Additional file

Additional file 1: **Figure S1.** Flow chart. **Figure S2.** Levels of CSF-PCT (a), serum PCT (b), CSF-CRP (c), serum PCT (d), CSF-leukocyte (e), and CSF-protein (f) in Gram-negative and Gram-positive BM. **Table S1.** Pathogens of patients with definite diagnoses of BM. Table S2. Type of BM. **Table S3.** Baseline characteristics. Table S4. Predictive performance of CSF-PCT in Gram-negative BM. (DOC 452 kb)

## Abbreviations

BM: Bacterial meningitis
CRP: C-reactive protein
CSF: Cerebrospinal fluid
N-ICU: Neurological intensive care unit
PCT: Procalcitonin
