# Supplementary material for: Cerebrospinal fluid procalcitonin predicts Gram-negative bacterial meningitis in patients with empiric antibiotic pretreatment
Source: Crit Care. 2019 Jan 23;23:21. doi: 10.1186/s13054-019-2318-8 (PMC6343261; doi:10.1186/s13054-019-2318-8)
Supplement: Supplementary file 1 — Figure S1. Flow chart. Figure S2. Levels of CSF-PCT (a), serum PCT (b), CSF-CRP (c), serum PCT (d), CSF-leukocyte (e), and CSF-protein (f) in Gram-negative and Gram-positive BM. Table S1. Pathogens of patients with definite diagnoses of BM. Table S2. Type of BM. Table S3. Baseline characteristics. Table S4. Predictive performance of CSF-PCT in Gram-negative BM. (DOC 452 kb) [file 13054_2019_2318_MOESM1_ESM.doc]

**References**

1. Schwarz S, Bertram M, Schwab S, Andrassy K, Hacke W. Serum procalcitonin levels in bacterial and abacterial meningitis. Crit Care Med. 2000;28:1828–1832.

2. Takahashi W, Nakada T, Abe R, Tanaka K, Matsumura Y, Oda S. Usefulness

of interleukin 6 levels in the cerebrospinal fluid for the diagnosis of bacterial

meningitis. Journal of Critical Care. 2014;29:693.e1-693.e6.

3. Tunkel AR, Hasbun R, Bhimraj A, Byers K, Kaplan SL, Michael Scheld W, et al. 2017 Infectious Diseases Society of America's Clinical Practice Guidelines for Healthcare-Associated Ventriculitis and Meningitis. Clin Infect Dis. 2017;64: e34–e65.

4. Jackson WL Jr, Shorr AF. Noscominal bacterial meningitis. N Engl Med. 2010;362:1346.

**Supplementary materials**

**Figure S1** Flow chart.

**Figure S2** Levels of CSF- PCT (a), Serum-PCT (b), CSF- CRP (c), Serum-PCT (d), CSF- Leukocyte (e), CSF- Protein (f) in Gram-negative and Gram-positive BM.

**Table S1** Pathogens of patients with definite diagnoses of BM.

**Table S2** Type of BM.

**Table S3** Baseline characteristics.

**Table S4** Predictive performance of CSF-PCT in Gram-negative BM.

**225 patients with suspected**

**meningitis/encephalitis**

**Clinical evaluation and diagnosis**

- **114 patients with TBM, VM/E, AIE**
- **21 patients excluded because of**

**unclear final diagnosis**

**90 patients with a definite**

**diagnosis of BM**^[1,2]^

**Age, mean (****±SD) : 44.2 ± 15.8**

**Male, n (%): 68 (75.6)**

**GCS , (IQR): 10.5 (7.8, 14.0)**

**Negative CSF culture**

**and Gram stain**

**(n = 31)**

**Gram+ BM**

**(n = 33)**

**Gram- BM**

**(n = 26)**

**Figure S1.** Flow chart.

The diagnosis of BM was based on compatible clinical features and one of three criteria, (i) positive CSF culture, (ii) negative CSF culture but with identification of bacteria by either CSF Gram staining or blood culture, or (iii) CSF white cell count of 500/mm^3^ or higher and rapid improvement after antibacterial therapy despite negative CSF and blood culture results. BM, bacterial meningitis; TBM, tuberculous meningitis; VM/E, viral meningitis/encephalitis; AIE, autoimmune encephalitis.


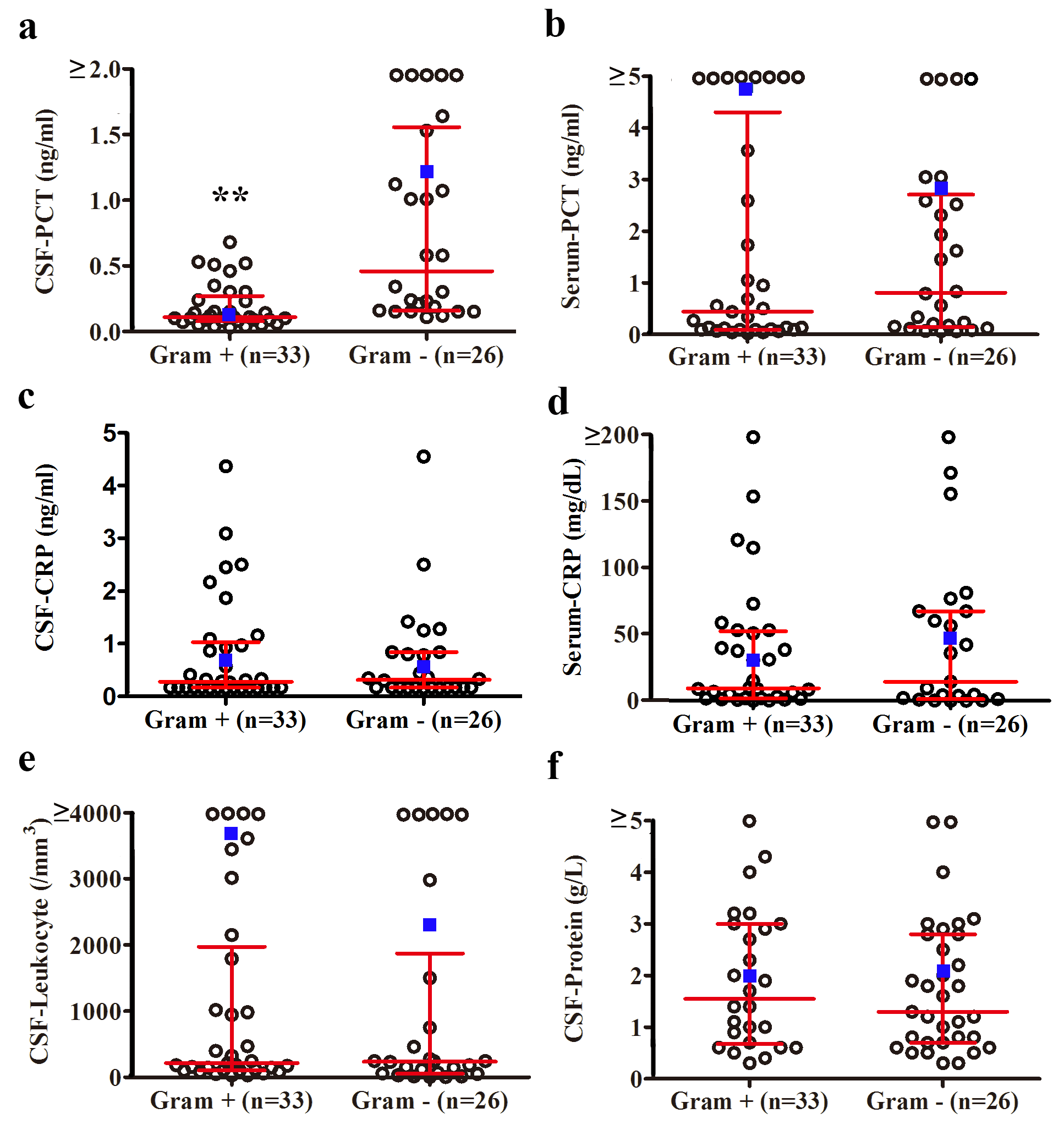


**Figure S2.** Levels of CSF- PCT (a), Serum-PCT (b), CSF- CRP (c), Serum-PCT (d), CSF- Leukocyte (e), CSF- Protein (f) in Gram-negative and Gram-positive BM. The red lines and blue squares correspond to the group interquartile ranges and averages, respectively. **, P＜ 0.001.

**Table S1.** Pathogens of patients with definite diagnoses of BM.

| **Pathogen** | **Number of patients** |
| --- | --- |
| Gram-positive BM | 33 |
| Staphylococcus haemolyticus | 4 |
| Enterococcus faecium | 2 |
| Streptococcus pneumoniae | 6 |
| Staphylococcus aureus | 3 |
| Staphylococcus epidermidis | 5 |
| Listeria monocytogenes | 3 |
| Staphylococcus capitis | 1 |
| Viridans streptococcus | 1 |
| Enterococcus faecalis | 1 |
| Staphylococcus saprophyticus | 1 |
| Unknown^a^ | 6 |
| Gram-negative BM | 26 |
| Klebsiella pneumoniae | 5 |
| Acinetobacter baumannii | 5 |
| Pseudomonas aeruginosa | 4 |
| Escherichia coli | 3 |
| Klebsiella oxytoca | 2 |
| Acinetobacter junii | 1 |
| Bacteroides fragilis | 1 |
| Burkholderia cepacia | 1 |
| Unknown^a^ | 4 |

^a^ positive Gram stain but negative CSF culture.

**Table S2.** Type of BM.

| **Type of BM**^[3]^ | **Number of patients** |
| --- | --- |
| Community-acquired BM | 25 |
| Streptococcus pneumoniae | 6 |
| Klebsiella pneumoniae | 5 |
| Staphylococcus aureus | 2 |
| Listeria monocytogenes | 3 |
| Gram-positive but negative CSF culture | 6 |
| Gram-negative but negative CSF culture | 3 |
| Nosocomial BM^[4]^ | 34 |
| Staphylococcus haemolyticus | 4 |
| Pseudomonas aeruginosa | 4 |
| Acinetobacter junii | 1 |
| Bacteroides fragilis | 1 |
| Staphylococcus epidermidis | 5 |
| Staphylococcus capitis | 1 |
| Viridans streptococcus | 1 |
| Enterococcus faecium | 2 |
| Acinetobacter baumannii | 5 |
| Staphylococcus saprophyticus | 1 |
| Staphylococcus aureus (MRSA) | 1 |
| Escherichia coli | 3 |
| Klebsiella oxytoca | 2 |
| Burkholderia cepacia | 1 |
| Enterococcus faecalis | 1 |
| Gram-negative but negative CSF culture | 1 |

CSF, cerebrospinal fluid.

**Table S3.** Baseline characteristics.

|  | **Total**  **(n = 59)** | **Gram-positive**  **(n = 33)** | **Gram-negative**  **(n = 26)** | ***P* value** |
| --- | --- | --- | --- | --- |
| Age, y | 46.5 ± 14.9 | 45.8 ± 13.0 | 47.4 ± 17.3 | 0.69 |
| Male, n(%) | 45 (76.3) | 27 (81.8) | 18 (69.2) | 0.26 |
| Fever, n(%) | 54 (91.5) | 31 (93.9) | 23 (88.5) | 0.78 |
| Headache, n(%) | 40 (67.8) | 23 (69.7) | 17 (65.4) | 0.73 |
| Seizure, n(%) | 14 (23.7) | 7 (21.2) | 7 (26.9) | 0.61 |
| Neck stiffness, n(%) | 47 (79.7) | 27 (81.8) | 20 (76.9) | 0.64 |
| Mental symptoms, n(%) | 23 (39.0) | 13 (39.4) | 10 (38.5) | 0.94 |
| Consciousness impairment, n(%) | 40 (67.8) | 21 (63.6) | 19 (73.1) | 0.44 |
| Vomiting, n(%) | 28 (47.5) | 14 (42.4) | 14 (53.8) | 0.38 |
| Focal neurological symptoms, n(%) | 16 (27.1) | 11 (33.3) | 5 (19.2) | 0.23 |
| Pneumonia, n(%) | 46 (78.0) | 26 (78.8) | 20 (76.9) | 0.86 |
| Sepsis, n(%) | 25 (42.4) | 13 (39.4) | 12 (46.2) | 0.60 |
| Glasgow coma scale score, n(%) |  |  |  | 0.51 |
| 9-15 score | 39 (66.1) | 23 (69.7) | 16 (61.5) |  |
| 3-8 score | 20 (33.9) | 10 (30.3) | 10 (38.5) |  |
| Corticosteroids treatment, n(%) | 20 (33.9) | 12 (36.4) | 8 (30.8) | 0.65 |
| Duration of empiric antibiotic pretreatment, d | 13.0 (7.0-19.0) | 13.0 (7.5-17.5) | 12.5 (5.8-21.0) | 0.75 |
| Time from onset to PCT test, d | 13.0 (7.0-21.0) | 13.0 (7.5-18.0) | 14.0 (7.0-22.3) | 0.48 |

CSF, cerebrospinal fluid; PCT, procalcitonin.

**Table S4.** Predictive performance of CSF-PCT in Gram-negative BM.

| **CSF-PCT Values** | **Cutoff** | **Sensitivity** | **Specificity** | **PPV** | **NPV** | **+LR** | **-LR** | **ACC** |
| --- | --- | --- | --- | --- | --- | --- | --- | --- |
| Optimal screen values^a^ | ≥ 0.11 | 96.2% | 54.6% | 63.2% | 90.5% | 2.2 | 0.1 | 74.6% |
| Optimal diagnostic values^b^ | ≥ 0.58 | 42.2% | 97.0% | 91.7% | 68.1% | 1.7 | 0.1 | 76.3% |
| Maximum accuracy (Youden Index) | ≥ 0.14 | 92.3% | 63.6% | 66.7% | 91.3% | 14.0 | 0.6 | 80.0% |

Abbreviations: PPV = positive predictive value; NPV = negative predictive value;

+LR = positive likelihood ratio; -LR = negative likelihood ratio; ACC = accuracy.

^a^Lowest value with sensitivity and NPV > 90%.

^b^Highest value with specificity and PPV > 90% when available.
